# **“**Anything that would help is a positive development”: feasibility, tolerability, and user experience of smartphone-based digital phenotyping for people with and without type 2 diabetes

**DOI:** 10.1186/s44247-024-00116-6

**Published:** 2024-09-12

**Authors:** A. M. McInerney, N. Schmitz, M. Matthews, S. S. Deschênes

**Affiliations:** 1 School of Psychology, University College Dublin, Belfield, Dublin 4, Ireland; 2 Department of Population-Based Medicine, University of Tübingen, Tübingen, Germany; 3 School of Computer Science, University College Dublin, Dublin, Ireland; 4 School of Psychology, University College Dublin, Dublin, Ireland

**Keywords:** Digital phenotyping, Ecological Momentary Assessment, Type 2 Diabetes, Feasibility, User experience, Tolerability

## Abstract

**Background:**

Digital phenotyping, the in-situ collection of passive (phone sensor) and active (daily surveys) data using a digital device, may provide new insights into the complex relationship between daily behaviour and mood for people with type 2 diabetes. However, there are critical knowledge gaps regarding its use in people with type 2 diabetes. This study assessed feasibility, tolerability, and user experience of digital phenotyping in people with and without type 2 diabetes after participation in a 2-month digital phenotyping study in Ireland. At study completion, participants rated methodology elements from “not a problem” to a “serious problem” on a 5-point scale and reported their comfort with the potential future use of digital phenotyping in healthcare, with space for qualitative expansion.

**Results:**

Eighty-two participants completed baseline. Attrition was 18.8%. Missing data ranged from 9–44% depending on data stream. Sixty-eight participants (82.9%) completed the user experience questionnaire (51.5% with type 2 diabetes; 61.8% female; median age-group 50–59). Tolerability of digital phenotyping was high, with “not a problem” being selected 76.5%—89.7% of the time across questions. People with type 2 diabetes (93.9%) were significantly more likely to be comfortable with their future healthcare provider having access to their digital phenotyping data than those without (53.1%), χ2 (1) = 14.01, *p* =  < .001. Free text responses reflected a range of positive and negative experiences with the study methodology.

**Conclusions:**

An uncompensated, 2-month digital phenotyping study was feasible among people with and without diabetes, with low attrition and reasonable missing data rates. Participants found digital phenotyping to be acceptable, and even enjoyable. The potential benefits of digital phenotyping for healthcare may be more apparent to people with type 2 diabetes than the general population.

**Supplementary Information:**

The online version contains supplementary material available at 10.1186/s44247-024-00116-6.

## Introduction

Digital phenotyping is a novel approach that leverages the vast amounts of data generated by digital devices, including smartphones and wearables, to offer unparalleled opportunities to observe and understand mental health and behaviour. Digital phenotyping has been defined as the “moment-by-moment quantification of the individual-level human phenotype in situ using data from personal digital devices” [1]. These data may be “passive” (e.g., GPS or call and text logs) which does not require engagement from the user, or “active” (e.g., daily diaries or ecological momentary assessments; EMAs) which does require active engagement from the user (e.g., providing a response to a question) [2]. By capturing rich and continuous streams of data, digital phenotyping can provide a reflection of the lived experience of a person as they go about their daily life. The potential power of digital phenotyping for advancing mental health research and treatment is underpinned by the idea that the data collected could be harnessed to create digital markers of mental ill-health, enhancing our ability to identify, monitor, and treat mental health issues [2]. One aspect of digital phenotyping that is receiving attention is its potential to tackle mental health challenges linked to chronic conditions [3, 4]. Mental health and mood in type 2 diabetes (T2D) is a potentially compelling area for exploration with digital phenotyping, due to the centrality of behaviour to T2D management and the potentially unique relationship between daily behaviour and mood for people with T2D.

Type 2 diabetes is a chronic condition characterised by the body’s inability to respond well to, or produce adequate, insulin [5]. A largely self-managed condition, it is associated with a substantial mental health burden [6]. Up to 30% of people with T2D experience depressive symptoms [7, 8], and as high as 36% experience diabetes distress [9], a construct encapsulating emotions related to diabetes’ unique problems. Despite substantial progress in medical care, recognising, preventing, and treating psychological distress in people with T2D remains a challenge [10, 11].

Recommended self-management behaviours for people with T2D can themselves be a significant source of stress and negative affect [12]. Stresses related to maintaining physical activity, diet, and blood glucose monitoring are among major components of diabetes-specific distress [13, 14]. Understanding the unique relationship between daily behaviour and mood in individuals with T2D is crucial for effective interventions and providing personalized care. Traditional longitudinal data collection methods, relying on retrospective self-reports with large gaps between collection occasions, offer limited insights into the dynamic associations between daily behaviours and mood in individuals with T2D. These methods fail to capture the nuanced and real-time interactions that might significantly impact mental health. Herein lies the potential of digital phenotyping—an approach that offers a more granular and ecologically valid understanding of the contextual, temporal relationship between behaviour and mood in people with T2D.

To realise this potential, and for digital phenotyping to be broadly adopted in diabetes-related research and clinical settings, its feasibility and tolerability as a data collection method for people with T2D must be evaluated. Mobile health and digital intervention applications (apps) have been shown to be acceptable to people with T2D [15, 16]. However, digital phenotyping presents a unique combination of challenges, including ethical and privacy concerns, high attrition, missing data, the actual and perceived burden experienced by participants, and technological limitations and connectivity issues pertaining to participants' devices. To our knowledge only two studies have examined feasibility of digital phenotyping in people with T2D [17, 18]. The first focused on engagement trajectories of using mHealth self-monitoring devices in people with T2D but did not utilize key digital phenotyping data streams, such as accelerometer, GPS, or EMA [18]. A second study evaluated adherence to digital phenotyping in people with and at risk of T2D but did not use a smartphone for recording passive streams, and used the MyFitnessPal app to log food, rather than an EMA protocol [17]. Both focused on engagement metrics, and thus it remains unclear if other aspects unique to smartphone-based active and passive digital phenotyping are tolerable to people with T2D. Additionally, conclusions about the tolerability and acceptability of digital phenotyping were limited in both studies by a lack of subjective user experience data.

Therefore, the objectives of the present study were as follows:

Feasibility:

- To ascertain the willingness of participants with T2D to engage in and sustain participation, while ensuring adequate collection of useable passive and active data, throughout a 2-month digital phenotyping study, involving people with and without T2D.
- To assess if study completion or missing data vary based on factors such as type of phone used (android v iPhone), T2D status, age, gender, employment status, marital status, and education.

Tolerability:

- To assess tolerability of specific aspects of digital phenotyping data collection methodology.
- To assess if tolerability varies based on age, employment status, T2D, and phone type.

User-experience:

- To assess and compare the subjective user experiences of people with and without T2D after completing a 2-month digital phenotyping study.

Finally, a critical factor in digital phenotyping’s potential as a future clinical tool is the proposed users’ level of comfort with their healthcare provider accessing and using digital phenotyping data. As such, this study also reports participants’ responses to being asked whether they think they would be comfortable with their healthcare provider, in a hypothetical future, having access to the kind of digital phenotyping data collected, and aims to compare responses between those with and without T2D.

## Method

### Participants

The sample are from a digital phenotyping study, The Smartphone, Behaviour, and Mood study, which took place in the Republic of Ireland between February and August 2021, with a goal of identifying digital phenotype correlates of psychological distress and examining the day-to-day social, affective, and behavioural processes related to psychological distress in adults with and without T2D. Given that the primary focus of The Smartphone, Behaviour, and Mood study was to assess feasibility and the practicality of implementation, including recruitment, sample size was not predetermined. Inclusion criteria included: (a) being over the age of 18 and under the age of 70; (b) living in the Republic of Ireland; (c) being able to read and write in English; and (d) owning a smartphone. Participants were recruited through convenience sampling through Diabetes Ireland, a nationwide diabetes charity; posts on social media; local radio; and articles and adverts in local newspapers throughout Ireland.

Ethical approval was obtained from University College Dublin’s Human Research Ethics Committee. Written informed consent was provided prior to enrolment.

### Procedure

Participants downloaded the Beiwe app [19] to their smartphones. The app passively collected GPS data, accelerometer data, and the number of calls and texts received and sent (android only). The app also delivered twice daily EMAs. In the morning, at 8 am, participants estimated their previous night's sleep duration and rated their sleep quality. An evening survey (7 pm) covered mood, number and positivity of social interactions, and amount of physical exercise. Both surveys had a 3-hour response window. Participants completed a battery of questionnaires at baseline (prior to downloading the app) and at a 1-month and 2-month follow-up. The baseline questionnaire collected demographic information and all questionnaires included a battery of psychosocial, lifestyle, and health questionnaires.

### User experience

An optional feedback questionnaire was included as part of the 2-month follow-up questionnaire battery at study completion to assess participants’ experience (see Supplementary file). Participants were asked to rate 5 different aspects of the data collection methods on the extent to which they felt each aspect was a problem, on a 5-point Likert scale, from 0 (“*not a problem*”) to 4 (“*serious problem*”). The aspects examined were: the length of the daily surveys; the length of the baseline and follow-up questionnaires; the frequency of the daily surveys; the type of phone sensor data being collected; and perceptions of the app’s effect on phone storage and battery. Participants were also provided space for open responses on other problems experienced and general feedback. Finally, participants were asked if they would feel comfortable with their healthcare provider having access to similar data, in a hypothetical future, with response options “Yes” or “No” and were given space to elaborate on their answers.

### Data analysis

Sample characteristics, presented as frequencies and percentages, were compared between those who completed the study and those who did not, and between those with and without diabetes among the completers. Data missingness was calculated as a percentage of EMA survey responses (with 2 in the morning, and 6 in the evening) received over 57 days. Passive phone sensor data missingness was calculated at the daily level, which considered data as missing if insufficient useable data was collected within 24 hours to generate a daily summary score (i.e., daily time spent at home). We investigated whether T2D status, gender, age, marital status, education level, and employment status varied depending on comfort level with potential future use and EMA data missingness. Additionally, we examined if data missingness varied by phone type. Answers to the tolerability questions were analysed for differences by age, employment status, T2D, and phone type. User experience open responses were analysed for differences based on T2D or tolerability. Independent samples *t*-tests and Pearson’s chi-square tests were used for continuous and categorical variables, respectively. Qualitative responses were grouped by content, with indicative quotes presented.

## Results

### Participants

**Fig. 1:**
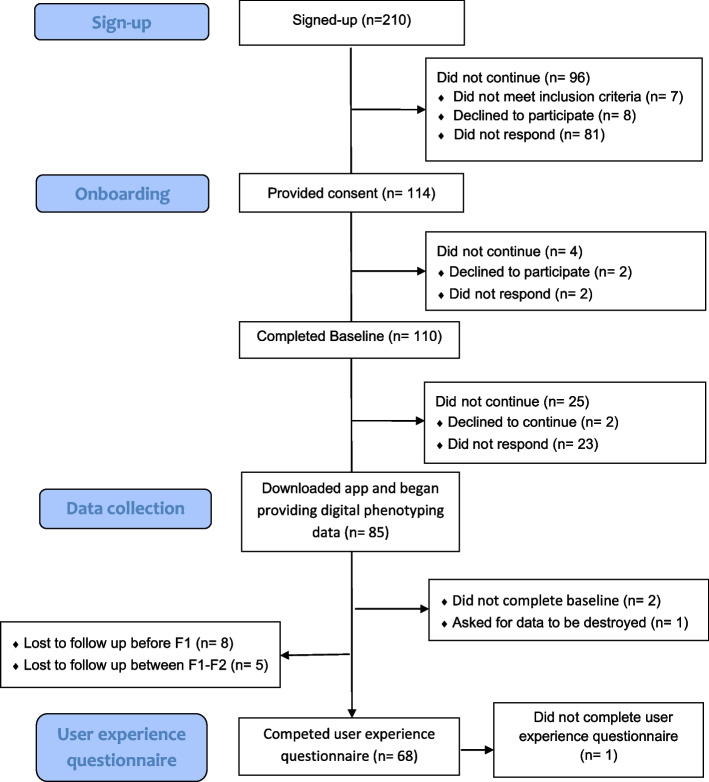
Participation Flowchart

Figure 1 presents the participation flowchart. Eighty-five participants downloaded the app, 82 completed the baseline assessment, and 68 participants (82.9% of the total sample; 92.1%, 35/38 of group with T2D; 75%, 33/44 of the group without T2D) completed the user experience questionnaire. The participants with T2D were more likely to be male, older, married, and disabled or retired than the participants without T2D. Table 1 presents the participant characteristics for those with T2D, without T2D, and the combined sample for those who completed the feedback questionnaire and those who did not.

**Table 1:** Participant characteristics

	Those who completed the feedback questionnaire							Those who did not complete feedback questionnaire		
	All *n* = 68		Diabetes *n* = 35		Control *n* = 33		*p* value*	All *n* = 14		*p* value#
	n	%	n	%	n	%		n	%	
Female	42	61.8%	17	48.6%	25	75.8%	< .05	9	63.3%	.903
Age							< .001			
18–29 years old	15	22.1%	0	0%	15	45.5%		4	28.5%	
30–39 years old	9	13.2%	0	0%	9	27.3%		4	28.6%	
40–49 years old	6	8.8%	5	14.3%	1	3%		1	7.1%	
50–59 years old	18	26.5%	15	42.9%	3	9.1%		2	14.3%	
60–70 years old	20	29.4%	15	42.9%	5	15.2%		3	28.1%	
Marital status							< .001			.353
Single (never married)	25	36.8%	4	11.4%	21	63.6%		4	28.6%	
Married or common-law partnership	38	55.9	27	68.6%	11	33.3%		7	50%	
Divorced, separated, or widowed	5	7.4%	4	11.4%	1	3%		3	21.4%	
Education							.258			.905
Secondary school or less	8	11.8%	5	14.3%	3	9.2%		2	14.3%	
Some post-secondary	16	23.5%	12	34.3%	4	12.1%		4	28.6%	
Completed Bachelor’s	22	32.4%	10	28.6%	12	36.4%		3	21.4%	
Masters or higher	22	32.4%	8	22.9%	14	42.4%		5	35.7%	
Employment status							< .05			.636
Employed (full or part-time)	35	51.5%	15	42.8%	20	60.6%		7	50%	
Student (full or part-time)	6	8.8%	0	0%	6	18.2%		1	7.1%	
Retired	14	20.6%	12	34.3%	2	6.1%		0	0%	
Disabled (not able to work)	6	8.8%	5	14.3%	1	3%		1	7.1%	
Unemployed (looking for work)	2	2.9%	0%	0%	2	6.1%		3	21.4%	
Unemployed (not looking for work)	5	7.4%	3	8.6%	2	6.1%		2	7.1%	
Ethnicity							.143			.618
White Irish	56	82.4%	32	91.4%	24	72.7%		10	71.4%	
Any other white background	9	13.2%	2	5.7%	7	21%		3	21.4%	
Black or black Irish- African	1	1.5%	0	0%	1	3%		0	0%	
Asian or Asian Irish-Chinese	1	1.5%	0	0%	1	3%		0	0%	
Asian or Asian Irish- Any other Asian background	1	1.5%	1	2.9%	0	0%		1	7.1%	
Self-rated health							< 0.05			.027
Excellent	8	11.8%	0	0%	8	24.2%		2	14.3%	
Very good	12	17.6%	4	11.4%	8	24.2%		8	57.1%	
Good	35	51.5%	24	68.6%	11	33.3%		3	21.4%	
Fair	9	13.2%	4	11.4%	5	15.2%		1	7.1%	
Poor	4	5.9%	3	8.5%	1	3%		0	0%	
BMI category							< .001			< .001
Underweight	3	4.4%	1	2.9%	2	6.1%		0	0%	
Normal weight	25	36.8%	5	14.3%	20	60.6%		8	57.1%	
Overweight	15	22.1%	9	25.7%	6	18.2%		4	28.6%	
Obese	22	32.3%	19	57.6%	3	9.1%		2	28.6%	
Missing	3	4.4%	1	2.9%	2	6.1%		0	0%	
	All		Diabetes		Control			All (diabetes sample = 3)		
Scores on psychological questionnaires	M	SD	M	SD	M	SD		M	SD	
Depression PHQ-9	6.4	6.1	6.8	6.6	5.8	5.5	0.512	6	4.7	.266
Anxiety GAD-7	5.3	5.4	4.9	5.1	5.8	5.7	0.454	4.1	3.6	.558
Diabetes Distress PAID-5	n/a	n/a	7	5.1	n/a	n/a		3.3	4.2	
Diabetes Stigma DSAS-2	n/a	n/a	48.2	18.4	n/a	n/a		24	1.7	

Due to missing data, reported sample sizes for BMI may not equal the total sample sizes

*PHQ-9* Patient Health Questionnaire, 9 items, total score range 0–27, *GAD-7* Generalized Anxiety Disorder Assessment, 7 items, total score range 0–21, *PAID-5* Problem Areas in Diabetes short form, 5 items, total score range 0–20, *DSAS-2* Type 2 Diabetes Stigma Assessment Scale, 19 items, total score range 19–95

^*^ = *p* values are for differences between people with and without diabetes. # = p values for differences between those who completed the feedback questionnaire and those who did not. Tests are Pearson’s Chi-Squared or independent samples for categorical and continuous variables, respectively

## Feasibility

### Attrition

As depicted in Fig. 1, 18.8% of those who downloaded the app completed the 2-month follow-up assessment. Reasons for discontinuation included one participant finding mood reflection distressing and another finding survey notifications bothersome. However, the majority did not provide reasoning but ceased responding to the research team and completing follow-up questionnaires. Only one participant who completed the 2-month follow-up declined to provide feedback. Those who completed the follow-up are compared to those who did not in terms of sociodemographic factors in Table 1.

#### Active and passive data

In the total baseline sample (*n* = 82), 37.8% used iPhones and 62.2% used Androids. These proportions were consistent for those who completed the 2-month follow-up (*n* = 69; 37.7% and 62.3%, respectively) and those who did not (*n* = 13; 38.5% and 61.5%, respectively).

Participants who completed follow-up were missing 40.4% of responses on the morning and 44% on the evening survey, compared to 83.4% on the morning and 85.9% on the evening survey for those who did not complete follow-up. For those who completed follow-up, iPhones had significantly more missing data than Androids for morning (70% vs 21.3%; *t*(66) = 8.623, *p* < 0.001) and evening (70.6% vs 26.8%; *t*(66) = 7.965, *p* < 0.001) surveys (see Table S1). There were no significant associations, for those who completed follow-up or for those who dropped out, between EMA missingness and T2D or sociodemographic covariates (Table S1).

With regards to the passive phone sensor data, participants who completed the study had 9.1% missing accelerometer and 14.4% missing GPS data, while those who dropped out had 49.9% missing accelerometer and 65.5% missing GPS. Call and text data could only be collected on Android phones. In the 51 android users, call and text data were missing on 40.2% of days. Those who completed the survey *(n* = 42) had 33.6% missing call data and those who did not (*n* = 9) had 70.8%. There were no significant associations between the type of phone used and data missingness for those who dropped out or for those who completed on accelerometer or GPS (see Table S1).

## Tolerability

Tolerability of passive and active data collection methods was high, with “*not a problem*” being selected 76.5%—89.7% of the time across the 5 questions (see Figure S1). There was evidence of a significant association between age and tolerability of the length of daily surveys (χ2(18) = 46.212, *p* < 0.001), indicating that older individuals were more inclined to answer “minor problem” (as opposed to “*not a problem*”) and between age and perceiving the app’s effect on phone storage or battery as a minor problem (χ2(36) = 56.645, *p* = 0.016), with younger people more likely to report this as a minor issue and older adults more likely to report it as a serious issue. There were no other significant associations between tolerability questions and age, employment, T2D, or phone type (Table S2).

### User experience

#### Free text responses: Other problems

Participants were then provided with free text space and the following prompt “Please feel free to provide us with any other problems you experienced during the study (optional)”. Of the 68 participants who completed the feedback questionnaire, *n* = 23 (33.8%) answered this question. Of these, *n* = 14 (60.9%) mentioned problems related to the app, citing issues such as the app being *“a bit temperamental”.*

A common issue experienced by participants was around accessing the surveys on the app, with participants relaying issues such as:

*“Sometimes surveys weren’t there despite sending a notification”,*

*“I would get a notification to do a survey and then when I clicked it, I’d just see a black screen until I reset the app and logged back in”.*

A further four responses reflected external (to the study methodology) issues that influenced the participant’s completion of daily surveys. For example, participants shared:

*“The only problem I had was I drive a taxi and work evenings and couldn’t always do the 7pm survey until later or by then it had disappeared off the app”*

*“I was not near Wi-Fi first thing in morning”.*

Three responses were positive or reflected that the participant had not experienced any problems, including “*enjoyed it”* and “*no problems other than my own ineptness with technology”.*

We compared those who provided positive or neutral responses to those who provided a negative response. There were no significant differences between those who answered the “any other problems” free text question and those who did not, or between those who answered negatively and those who answered positively/neutrally, in terms of tolerability for the five data collection questions (Table S3). There was a significant difference between people with and without T2D in their likelihood to give a negative response, (χ2(1) = 5.789, *p* = 0.016), with 45.5% of people with T2D giving a negative response compared to 91.6% of people without T2D. There was no significant difference between those who answered and those who did not in T2D (Table S3).

#### Free text responses: Other feedback

Participants were also provided with a second free text box and given the prompt “Please feel free to provide us with any other feedback you may have (optional).” Fourteen participants answered this question, *n* = 10 of whom had not answered the previous free text question. Eight (57.1%) of the 14 responses reflected positive experiences.

Participants expressed enjoyment in reflecting on their day when answering the EMAs:

*“I enjoyed taking part and counting up the number of interactions I had each day”*

*“The daily surveys were a useful tool for my own personal reflections, and being more mindful of how I was feeling each day”*

*“The study was simple and interesting, I managed to observe closely how much and how good is my sleep, how much I exercise and what should I work on”*

*“It was a real pleasure and relatively effortless, thank you”*

Participants also commented on their participation in light of the broader context of diabetes research and awareness:

*“It was interesting to take part in this study and hopefully more awareness about Diabetes Type 2 will happen.”*

*“I'm just glad to have participated in the research and I hope it will be of huge benefit down the line for all future research in to type 2 Diabetes. This is such a serious issue for the health of our nation going forward.”*

Three participants provided neutral reflections on their experience of taking part and thoughts on what may have impacted their behaviour or data, for example:

*“I also have asthma and a chronic pain condition which are other variables and so affect my quality of sleep and mood as well as ability to exercise”.*

*“The daily question on activity actually got me active as I had not been doing any exercise due to my sedentary job and sitting in a chair all day and every day”*

Three participants mentioned issues related to accessing surveys and the functioning of the app.

Significant differences were observed between respondents who provided negative responses to this question and those who did not regarding their responses to the tolerability questions concerning survey length (χ2(1) = 3.949, *p* = 0.47) and the frequency of daily surveys (χ2(1) = 3.949, *p* = 0.047). Sample sizes were small for these comparisons, with only one participant indicating a moderate problem among those who responded to both the tolerability questions and completed the open feedback question. There were no other significant associations between respondents and non-respondents, and those who responded negatively and those who did not, regarding T2D or tolerability (Table S3).

### Question About a Hypothetical Future

Participants were also asked to respond to the following hypothetical question: “We would like to ask you a hypothetical question (a question about a possible future). In the future, would you feel comfortable with your healthcare provider having access to information collected from your smartphone to better meet your needs and provide you with care?”.

Three free text responses were incongruent with the selected answer to the hypothetical question (e.g., “Yes, I would feel comfortable” combined with free text response “feels a little intrusive”) and therefore were removed from the analysis. The difference in positive responses between people with (93.9%) and without (53.1%) T2D was statistically significant (χ2(1) = 14.012, *p* < 0.001). A significant association also emerged concerning employment status (χ2(1) = 7.647,* p* = 0.006). Specifically, 88.2% of people who said they would not be comfortable were employed or in education (as opposed to retired, unemployed, or unable to work) compared with 50% of those who were comfortable. There were no other significant associations between feeling comfortable with the future use of such technology in healthcare and sociodemographic factors (Table S4).

### Hypothetical Future: Free text responses

Participants were also provided with free text space to elaborate on the reasoning behind their answer to the question about a hypothetical future. Thirty-seven participants completed this section.

People without T2D mentioned privacy concerns and lack of comfort with the data collected in their responses. Some participants without T2D also raised issues about the usefulness of digital phenotyping data and the conclusions that can be drawn from it. For example:

*“I am concerned about . . . possible use of my data to draw conclusions about me that might not be correct”*

*“phone data is limited in what it can actually tell you about a person's lived experiences”*

However, participants without T2D also recognised the potential of digital phenotyping to be advantageous for healthcare:

*“I think it would be a more insightful way to observe patients’ health data for the doctors”*

*“they'd have a better picture of the problem and give them context so they can better diagnose”*

*“The more data and information our doctors have the less they need to get from the patient who may not be able to describe their symptoms correctly or may forget something”*

Conversely, only one person with T2D provided a reason for not feeling comfortable with their future healthcare provider having access to digital phenotyping data *(“I can manage myself so far and happy enough”).*

Participants with T2D mentioned that the data may be *“insightful”, “useful”,* and an *“easier and better way to make a judgement”.* Some stated that they considered “*any risk well worth taking”* and that* “anything that would help is a positive development”.*

Many comments from people with T2D recognised the potential benefit for their healthcare, including:

*“You meet your doctor a couple of times per year, it’s hard to explain all issues in such a small window of opportunity, so that the doctor would have weekly/monthly data would made him more in tune with my issues”,*

*“If it helps in my care and healthcare, I am all for my GP having my answers”,*

*“I am quite ill in several different aspects, and no one seems to co-ordinate them really. I am frustrated at not getting well and the length of time it takes to see a relevant person (consultant etc.). If an app can help any of these problems for me and improve my health, I'd do it”,*

*“I assume it may help them address my condition better”.*

## Discussion

The present study assessed the feasibility, tolerability, and user experience of a 2-month digital phenotyping study in a cohort of adults, living in Ireland, with and without T2D. There was low attrition and reasonably low rates of missing data, except for EMA data among iPhone users. Overall, tolerability of the data collection method was very high in both people with and without T2D. Participants expressed enjoyment in taking part and answering daily surveys. However, some also mentioned technical issues with the digital phenotyping application and accessing surveys. People with T2D were significantly more likely than those without to provide positive or neutral responses (compared to negative) to the “any other problems” question and to feel comfortable with their healthcare provider having access to similar data in a hypothetical future, suggesting that people with T2D may represent a group for which the benefits of digital phenotyping are particularly apparent.

### Feasibility

This study was carried out by participants on a volunteer basis (i.e., without financial compensation) and during the COVID-19 pandemic, and thus, participant onboarding and troubleshooting had to be carried out remotely. Despite this, there was low attrition, with 81.2% of participants who downloaded the app, and 84.2% of those who completed baseline, completing the 2-month follow-up.

### Active data

For those who completed the study, data missingness ranged from 40% for the morning survey to 44% for the evening EMA survey. Both study completers and non-completers were more likely to answer morning surveys than evening, which is in line with other research [20]. Our rates of EMA survey completion (56%-60%) were lower than a pooled compliance rate of 75% in a meta-analysis of digital phenotyping studies with substance users [21]. However, 77% of included studies in this meta-analysis had a financial incentive, and the authors note that other studies did not explicitly state reimbursement. Incentives have been shown to positively influence EMA compliance [21, 22], along with higher time intervals between surveys and fewer daily surveys, which may have impacted responses [21]. It was also evident from participant feedback in the open responses that many issues were experienced with the app, where participants wanted to complete surveys, but were unable to, which may also have impacted response rates.

iPhones were significantly more likely to have missing EMA responses than Android phones. A study comparing iOS and Android users of an mHealth platform found android users provided more EMA responses (52.13 ± 67.64) than iOS users (35.59 ± 31), though this difference was not statistically significant [23]. While a meta-analyses of studies using Beiwe compared iOS and Android devices, they only did so for GPS and accelerometer, finding iOS to have significantly lower GPS non-collection (RR: 0.66 [95% CI: 0.45, 0.95]) [24]. To our knowledge, Beiwe EMA collection has not been compared between iPhone and Android smartphones. Further research is needed to determine if users of different smartphone operating systems respond to EMA studies differently.

Tolerability of daily EMA surveys was high amongst our sample, with 89.7% and 82.4% selecting “*not a problem*” for survey length and frequency, respectively. Only one response of “*somewhat serious problem*” was recorded for frequency of EMA surveys. The high level of tolerability indicates that surveys of sufficient length and frequency to capture a reasonably thorough subjective picture of components of a person’s daily mood and behaviour are tolerable to participants with and without T2D. There was a significant association between age and two of the tolerability questions related to the active data collection. Older participants were more likely to find the length of the daily surveys to be a minor a problem. EMA surveys that are short in duration and frequency can improve compliance in elderly participants [22]. The EMA surveys (comprising sliding scale and drop-down menus) were designed and piloted to be competed in 1–3 min. However, these may have felt laborious depending on a person’s comfort with technology. It is recommended that older participants receive training on EMA equipment to reduce burden and increase compliance [22], which was not possible to do in-person during this study. Older participants were also more likely to report the app’s effect on phone battery and storage to be a serious issue (the only issue to receive a response of “serious issue”). The Beiwe app was developed to sample GPS periodically (for 90 s every 1000 s) to limit the draining of battery [1] but there may always be some impact on battery life of such intensive passive phone sensor collection.

### Passive data

The digital phenotyping application Beiwe collected rich, longitudinal data across a wide range of smartphone passive sensors (GPS, accelerometer, call and text logs). There were fewer instances of missing data for passive data streams compared to EMA (9.1% for accelerometer, 14.4% for GPS, and 33.6% for call data), as would be expected due to it not requiring participant action. The unobtrusive nature of passive digital phenotyping data is one of its primary advantages [4]. Passive data is particularly appealing to psychological and behavioural researchers as it may provide an avenue for accessing ecologically valid, objective measures of important features of human behaviour that have not been easily reached traditionally, such as daily sleep, movement, or emotional state [16]. Free text responses indicate that, while there were some technical issues with the application in relation to EMA surveys, this intensive passive data was collected with minimal participant burden.

### Technical issues

Participants reported experiencing several problems while taking part in the study. As discussed above, the app’s effect on phone storage/battery was the only problem in the survey to receive answers of “serious problem” (4% of respondents, 3/68). Free text responses indicated that issues with the functionality of the app were experienced by some participants, including the app “glitching”, showing a blank screen, or surveys not being accessible after receiving a notification. However, issues with the app and surveys were only mentioned by 16 individual participants across both free text questions. This represents 24% of those who completed the feedback questionnaire. Given that negativity bias is a well-documented phenomenon in open-ended questions, with those who are dissatisfied more likely to respond than those who are more satisfied [25], these technical issues appear to have been mentioned by a relatively small proportion of study participants.

### Privacy concerns

Privacy concerns have been highlighted as a potential barrier to participation in digital phenotyping studies [26]. In this study, when asked about the type of phone sensor data (i.e., the passive data) being collected, 94% of people with and 79% of people without T2D indicated that this was “not a problem”. This corresponds with findings from a study assessing digital phenotyping acceptability with US veterans, where only 6% (4/67) of participants felt the data being collected “violated their privacy” [27]. Regarding healthcare provider access to this data in the future, 6 participants expressed concerns related to “*intrusiveness”*. An increase in international media coverage of data breaches in mobile apps [28], fears around COVID-19 tracing using GPS [29], and in a 2021 cyberattack on the Irish Health Service [30], are likely to have raised wariness of apps collecting passive data. However, only 9% of respondents raised these concerns.

While these findings may appear to provide support for privacy concerns not being an inhibitory issue for digital phenotyping studies, the sample likely exhibits inherent bias. There may have been a selection bias, where those who responded to recruitment advertisements may have been less concerned about smartphone privacy issues. Moreover, participants received a very detailed information sheet prior to enrolling, outlining the data to be collected and the risk of a data breach. Participants who chose not to continue or who dropped out during data collection may also have had more concerns than those who completed data collection and the feedback questionnaire. Future research should use survey and qualitative methods such as focus groups to assess privacy and security concerns in the general population to get a true estimate of views of digital phenotyping technology in research and clinical settings.

### Positive experiences of participation

Participants also expressed enjoyment in participating and answering surveys, describing it as “*a real pleasure and relatively effortless*”, and *“simple and interesting”.* Some participants noted positive impacts on their behaviour and thoughts, feeling compelled to be more “*active*”, or allowing them to be “*more mindful”* of how they feel, or providing clarity on what to “*work on*”. “Reactive effects” [31], changes in a person’s behaviour due to the method of measuring the behaviour, are well documented in research that uses diary keeping [32]. Research on the effects of participation in digital phenotyping studies and answering EMAs on participant behaviour is lacking, though a commonly used tool for measuring activity, the pedometer, has been consistently shown to impact behaviour [33, 34]. There is evidence that participating in any health psychology research alters participants feelings, thoughts, and behaviours and that this is biasing an unknown amount of research published each year [35]. Research is needed to determine the effect on behaviour of measurement that requires daily self-report in digital phenotyping studies.

Hopes for their participation making a difference were also reflected in some participant responses *(“I hope it will be of huge benefit down the line for all future research in to type 2 Diabetes”).* This corresponds with findings from a study using semi-structured interviews to explore participants experiences after a 2-week EMA study, where the theme of “making a difference” emerged along with the themes of “enjoying the experience”, “self-reflection”, and “routine” [36]. Moreover, some participant responses appeared to recognise both their participation and digital phenotyping in general as adding to the progress of a more holistic approach to T2D research and healthcare. Despite the recognised, critical importance of psychosocial factors in diabetes care [37], a multinational evaluation found that only 8% of diabetes research funds allocated in the five years leading up to 2016 went to studies with a psychosocial focus [38]. Given the complex nature of living with and managing T2D, progress in promoting healthier, happier lives for people with T2D likely requires research and care that is multifaceted and has a psychosocial approach.

### Hypothetical future

After taking part in a 2-month digital phenotyping study, 75.8% of our respondents indicated that they would be comfortable with their healthcare provider having access to the type of digital phenotyping data collected in this study. Of those who indicated that they were not comfortable and elaborated on their answer, all but one reflected concern with privacy. The potential “*insightfulness*” of the data collected for healthcare providers was frequently mentioned.

People with T2D were significantly more comfortable with the hypothetical scenario described. Many saw the benefits outweighing any potential danger (e.g., *“any risk well worth taking”).* The value of this data to help their doctor better understand any issues they were having and the benefit of “*anything that would help*” them manage their diabetes and health was referred to by many of the participants with T2D. Given the self-managed nature of diabetes, people with diabetes are accustomed to monitoring their daily behaviour [37]. People with T2D recognise that healthcare professionals play an active and integral role in the emotions surrounding living with T2D and helping them to manage their condition [39]. Providing their healthcare provider with information on their experiences of high and low blood sugar, daily management, and other health behaviours is customary during T2D clinic appointments. The greater comfort with the idea of a healthcare provider having access to digital phenotyping data among people with T2D might reflect their heightened recognition of its benefits compared to the general population.

#### Strengths and Limitations

The present study had several strengths. To our knowledge, this study is the first to collect subjective user experience data from a digital phenotyping study for people with T2D. Subjective, open responses allow for a deeper exploration of thoughts, feelings, issues, and benefits. The present sample with diabetes was largely representative of the general Irish population with diabetes. For example, compared to people with diabetes in the baseline wave of the Irish Longitudinal Study on Aging (TILDA), a nationally representative prospective study of adults aged 50 and over in the Republic of Ireland, the demographics of our sample were similar, except for educational attainment, where our sample were more likely to have third level education (TILDA participants with diabetes were 66.4 years old, 42.4% female, 91% born in Ireland, 63.7% married, and 22.1% had post-secondary school education) [40]. The Beiwe application collected data across a number of passive streams and sent 2 EMAs daily, over 2-months. The findings are therefore, considering the intensiveness of the data collection approach in this study, meaningful for future research in the field. However, there are also several limitations that must be considered. The study sample was predominately white, highly educated, and over the age of 50, and so findings may not be generalizable to other populations. The sample without T2D were younger, and more likely to be female, single, and have third level education or higher than the participants with T2D, which may have contributed to the differences in experiences and viewpoints between the groups found in this study. The study sample consisted of people who responded to recruitment information on a smartphone based digital phenotyping study and were comfortable enough with the data collected to participate. Findings around privacy concerns and value of such data are likely to be positively biased. It is also possible that those who dropped out during the 2-month study, and did not complete the feedback questionnaire, had a worse experience than those who completed it. Finally, the administration of the feedback questionnaire through an online survey platform did not allow for truly in-depth expansion or follow up questions. Future research could use focus groups or semi-structured interviews with those who have participated in digital phenotyping studies and those who have not, to further explore issues, benefits, and barriers to participation.

## Conclusion

Two-months of intensive digital phenotyping was feasible in a sample of people with and without T2D. However, further research is needed to fully understand barriers to sustained participation and the factors that influence missing data. Both passive and active data collection was acceptable to participants, and many expressed enjoying the experience of participating. In particular, people with T2D recognised the potential for digital phenotyping data to improve their condition management and their doctor’s ability to provide care. The potential for the use of digital phenotyping clinically is only as strong as its acceptability amongst the proposed users. The user experience data from this study suggests that the benefits of digital phenotyping in mental and physical healthcare may be particularly evident to people with T2D, over and above the broader general population.

## Supplementary Information

Supplementary Material 1.

Supplementary Material 2.

## Data Availability

Authors have made data (i.e., tables of quotes from participants) available on Open Science Framework (10.17605/OSF.IO/T94FN, https://osf.io/t94fn/?view_only=433369822544476a849a079729e2d866).
