# Supplementary material for: “Anything that would help is a positive development”: feasibility, tolerability, and user experience of smartphone-based digital phenotyping for people with and without type 2 diabetes
Source: BMC Digit Health. 2024 Sep 12;2(1):55. doi: 10.1186/s44247-024-00116-6 (PMC11390910; doi:10.1186/s44247-024-00116-6)
Supplement: Supplementary file 1 — Supplementary Material 1. [file 44247_2024_116_MOESM1_ESM.docx]

**Supplementary file 1**

This document contains supplementary tables TableS1, TableS2, TableS3 and TableS4. It also contains a copy of the feedback questionnaire provided at study completion.

TableS1

**Missing data.** This table contains Independent samples t tests and Pearson Chi-Square tests comparing data missing levels on sociodemographic covariates and phone type, for those who completed the study and those who did not.

|  |  | Mean | sd | t/ χ | df | Sig (2-tailed) |
| --- | --- | --- | --- | --- | --- | --- |
| Those who completed feedback (n = 68) | | | | | | |
| Morning EMA missing | | | | | | |
| Phone # | iPhone (n=26) | 70% | 21.2% | 8.623 | 66 | **<.001** |
|  | Android (n=42) | 21.3% | 23.5% |  |  |  |
| T2D # | Yes (n=3) | 36.3% | 31.7% | .978 | 66 | .332 |
|  | No (n=11) | 44.1% | 34% |  |  |  |
| Gender # | Male (n=25) | 35.4% | 29.5% | .978 | 66 | .332 |
|  | Female (n=42) | 41.2% | 33.9% |  |  |  |
| Age * | 18-29 years old (n=15) | 44.4% | 33.8% | 54.174 | 52 | .391 |
|  | 30-39 years old (n=9) | 49.6% | 33.6% |  |  |  |
|  | 40-49 years old (n=7) | 51.6% | 33.6% |  |  |  |
|  | 50-59 years old (n=18) | 30.4% | 34.1% |  |  |  |
|  | 60-70 years old (n=20) | 37.2% | 30.6% |  |  |  |
| Education * | Secondary school or less | 29.6% | 19.7% | 157.314 | 156 | .455 |
|  | Some post-secondary | 24.3% | 19.9% |  |  |  |
|  | Completed Bachelor’s | 46.2% | 36.4% |  |  |  |
|  | Masters or higher | 48.8% | 36.6% |  |  |  |
| Employment* | Employed (full or part-time) (n=35) | 42.7% | 36.3% | 53.380 | 52 | .421 |
|  | Student (full or part-time)(n=6) | 33.5% | 28.4% |  |  |  |
|  | Retired (n=14) | 33.5% | 28.4% |  |  |  |
|  | Disabled (not able to work)(n=6) | 24.3% | 27.5% |  |  |  |
|  | Unemployed (looking for work)(n=2) | 80.3% | 13% |  |  |  |
|  | Unemployed (not looking for work)(n=5) | 37.2% | 21.3% |  |  |  |
| Marital status * | Single (never married) (n=25) | 46.9% | 35.3% | 187.718 | 208 | .840 |
|  | Married or common-law partnership (n=38) | 37.9% | 31.8% |  |  |  |
|  | Divorced, separated, or widowed (n=5) | 20.6% | 19.6% |  |  |  |
| Evening EMA missing | | | | | | |
| Phone# | iPhone (n=26) | 70.6% | 16.9% | 7.965 | 66 | **<.001** |
|  | Android (n=42) | 26.8% | 24.6% |  |  |  |
| T2D # | Yes (n=3) | 39.9% | 30.4% | 1.036 | 66 | .304 |
|  | No (n=11) | 47.6% | 30.8% |  |  |  |
| Gender # | Male (n=25) | 41.1% | 27.1% | 1.036 | 66 | .304 |
|  | Female (n=42) | 43.6% | 31.9% |  |  |  |
| Age * | 18-29 years old (n=15) | 47.8% | 28.1% | 52.037 | 50 | .395 |
|  | 30-39 years old (n=9) | 54.1% | 32.2% |  |  |  |
|  | 40-49 years old (n=7) | 50.8% | 29.9% |  |  |  |
|  | 50-59 years old (n=18) | 33.3% | 34.7% |  |  |  |
|  | 60-70 years old (n=20) | 42.6% | 28.2% |  |  |  |
| Education * | Secondary school or less (n=8) | 36% | 21.3% | 161.178 | 150 | .252 |
|  | Some post-secondary (n=16) | 30.2% | 22.1% |  |  |  |
|  | Completed Bachelor’s (n=22) | 49.6% | 33.5% |  |  |  |
|  | Masters or higher (n=22) | 49.9% | 33.6% |  |  |  |
| Employment* | Employed (full or part-time) (n=35) | 46.7% | 33.4% | 52.684 | 50 | .371 |
|  | Student (full or part-time)(n=6) | 42.1% | 29.8% |  |  |  |
|  | Retired (n=14) | 39% | 26.7% |  |  |  |
|  | Disabled (not able to work)(n=6) | 28.3% | 27.8% |  |  |  |
|  | Unemployed (looking for work)(n=2) | 81.6% | 6.2% |  |  |  |
|  | Unemployed (not looking for work)(n=5) | 39% | 24.8% |  |  |  |
| Marital status * | Single (never married) (n=25) | 51.2% | 31.6% | 163.393 | 200 | .973 |
|  | Married or common-law partnership (n=38) | 41.1% | 30% |  |  |  |
|  | Divorced, separated, or widowed (n=5) | 23.2% | 21.6% |  |  |  |
| Accelerometer missing | | | | | | |
| Phone # | iPhone (n=26) | 9.3% | 20.3% | .047 | 66 | .963 |
|  | Android (n=42) | 9.1% | 13.1% |  |  |  |
| GPS missing | | | | | | |
| Phone # | iPhone (n=26) | 10.9% | 18.5% | -1.026 | 66 | .309 |
|  | Android (n=42) | 16.8% | 25.3% |  |  |  |
| Those who dropped out (n = 13) & did not complete feedback (n =1) | | | | | | |
| Morning EMA missing | | | | | | |
| Phone # | iPhone (n=5) | 85.8% | 11 | .417 | 12 | .684 |
|  | Android (n=9) | 81% | 24 |  |  |  |
| T2D # | Yes (n=3) | 93.6% | 5.6% | -1.072 | 12 | .305 |
|  | No (n=11) | 79.7% | 21.6% |  |  |  |
| Gender # | Male (n=5) | 74.2% | 31.7% | -1.210 | 12 | .250 |
|  | Female (n=9) | 87.4% | 8.5% |  |  |  |
| Age * | 18-29 years old (n=3) | 89.1% | 11.8% | 9.644 | 11 | .563 |
|  | 30-39 years old (n=4) | 86.4% | 8.3% |  |  |  |
|  | 40-49 years old (n=1) | 85.1% |  |  |  |  |
|  | 50-59 years old (n=2) | 56.6% | 49% |  |  |  |
|  | 60-70 years old (n=3) | 86% | 16.1% |  |  |  |
| Education * | Secondary school or less (n=2) | 92.1% | 11.2% | 32.200 | 33 | .507 |
|  | Some post-secondary (n=4) | 80.7% | 11.4% |  |  |  |
|  | Completed Bachelor’s (n=3) | 65.8% | 39% |  |  |  |
|  | Masters or higher (n=5) | 90.7% | 7.7% |  |  |  |
| Employment* | Employed (full or part-time) (n=7) | 71.2% | 22.7% | 14.000 | 11 | .233 |
|  | Student (full or part-time) (n=1) | 84.2% |  |  |  |  |
|  | Retired (n=0) | 0% |  |  |  |  |
|  | Disabled (not able to work) (n=1) | 100% |  |  |  |  |
|  | Unemployed (looking for work) (n=3) | 96.5% | 4.6% |  |  |  |
|  | Unemployed (not looking for work) (n=2) | 93% | 4.9% |  |  |  |
| Marital status * | Single (never married) (n=4) | 83.8% | 10% | 56.000 | 44 | .106 |
|  | Married or common-law partnership (n=7) | 88.6% | 11.6% |  |  |  |
|  | Divorced, separated, or widowed (n=3) | 67.6% | 39.5% |  |  |  |
| Evening EMA missing | | | | | | |
| Phone # | iPhone (n=5) | 91.6% | 7.1% | .887 | 12 | .393 |
|  | Android (n=9) | 81.3% | 25% |  |  |  |
| T2D # | Yes (n=3) | 97.1% | 3.6% | -1.154 | 12 | .271 |
|  | No (n=11) | 81.7% | 22.3% |  |  |  |
| Gender # | Male (n=5) | 76.6% | 32.9% | -1.154 | 12 | .271 |
|  | Female (n=9) | 89.7% | 9.2% |  |  |  |
| Age * | 18-29 years old (n=3) | 89.1% | 11.9% | 11.822 | 10 | .297 |
|  | 30-39 years old (n=4) | 86.8% | 8.2% |  |  |  |
|  | 40-49 years old (n=1) | 93.23% |  |  |  |  |
|  | 50-59 years old (n=2) | 58.8% | 55.8% |  |  |  |
|  | 60-70 years old (n=3) | 91.8% | 8.8% |  |  |  |
| Education * | Secondary school or less (n=2) | 92.1% | 11.2% | 33.950 | 30 | .283 |
|  | Some post-secondary (n=4) | 86.9% | 11% |  |  |  |
|  | Completed Bachelor’s (n=3) | 65% | 40.6% |  |  |  |
|  | Masters or higher (n=5) | 92.7% | 6.6% |  |  |  |
| Employment* | Employed (full or part-time) (n=7) | 74.3% | 25% | 14.000 | 10 | .173 |
|  | Student (full or part-time) (n=1) | 84.2% |  |  |  |  |
|  | Retired (n=0) | 0% |  |  |  |  |
|  | Disabled (not able to work) (n=1) | 100% |  |  |  |  |
|  | Unemployed (looking for work) (n=3) | 98.8% | 1% |  |  |  |
|  | Unemployed (not looking for work) (n=2) | 94.9% | 2.7% |  |  |  |
| Marital status * | Single (never married) (n=4) | 83.8% | 10% | 45.250 | 40 | .262 |
|  | Married or common-law partnership (n=7) | 92% | 7.7% |  |  |  |
|  | Divorced, separated, or widowed (n=3) | 70.2% | 44.1% |  |  |  |
| Accelerometer missing | | | | | | |
| Phone # | iPhone (n=5) | 57.6% | 39.7% | .783 | 12 | .449 |
|  | Android (n=9) | 40.4% | 39.2% |  |  |  |
| GPS missing | | | | | | |
| Phone | iPhone (n=5) | 70.2% | 32.9% | .732 | 12 | .478 |
|  | Android (n=9) | 55.6% | 37.2% |  |  |  |

Notes: # = Independent samples t-test; * = Pearson Chi-Square. Significant p values are highlighted in bold. Abbreviations: *sd* = Standard Deviation; *df* = Degrees of Freedom; Type 2 Diabetes

Table S2

This table contains Pearson Chi-Square tests for relationships concerning tolerability feedback questions.

| Tolerability feedback questions (n=68) | | | | | |
| --- | --- | --- | --- | --- | --- |
|  |  | % not a problem | χ | df | Sig (2-sided) |
| Feedback1 | | | | | |
| Age | 18-29 years old (n=15) | 100% | 46.212 | 18 | **<.001** |
|  | 30-39 years old (n=9) | 88.9% |  |  |  |
|  | 40-49 years old (n=6) | 50% |  |  |  |
|  | 50-59 years old (n=18) | 88.9% |  |  |  |
|  | 60-70 years old (n=20) | 95% |  |  |  |
| Employment | Employed (full or part-time) (n=35) | 88.6% | .927 | 2 | .629 |
|  | Student (full or part-time) (n=6) | 100% |  |  |  |
|  | Retired (n=14) | 92.9% |  |  |  |
|  | Disabled (not able to work) (n=6) | 83.3% |  |  |  |
|  | Unemployed (looking for work) (n=2) | 100% |  |  |  |
|  | Unemployed (not looking for work) (n=5) | 80% |  |  |  |
| T2D | Yes (n=36) | 83.3% | 3.460 | 2 | .177 |
|  | No (n=32) | 96.9% |  |  |  |
| Phone | iPhone (n=26) | 84.6% | 2.089 | 2 | .352 |
|  | Android (n=42) | 92.9% |  |  |  |
| Feedback 2 | | | | | |
| Age | 18-29 years old (n=15) | 73.3% | 39.640 | 27 | .055 |
|  | 30-39 years old (n=9) | 88.9% |  |  |  |
|  | 40-49 years old (n=6) | 50% |  |  |  |
|  | 50-59 years old (n=18) | 77.8% |  |  |  |
|  | 60-70 years old (n=20) | 95% |  |  |  |
| Employment | Employed (full or part-time) (n=35) | 74.3% | 7.736 | 3 | .052 |
|  | Student (full or part-time) (n=6) | 83.3% |  |  |  |
|  | Retired (n=14) | 92.9% |  |  |  |
|  | Disabled (not able to work) (n=6) | 100% |  |  |  |
|  | Unemployed (looking for work) (n=2) | 50% |  |  |  |
|  | Unemployed (not looking for work) (n=5) | 80% |  |  |  |
| T2D | Yes (n=36) | 80.6% | .932 | 3 | .818 |
|  | No (n=32) | 81.3% |  |  |  |
| Phone | iPhone (n=26) | 73.1% | 2.636 | 3 | .451 |
|  | Android (n=42) | 85.7% |  |  |  |
| Feedback 3 | | | | | |
| Age | 18-29 years old (n=15) | 73.3% | 31.838 | 27 | .238 |
|  | 30-39 years old (n=9) | 77.8% |  |  |  |
|  | 40-49 years old (n=6) | 50% |  |  |  |
|  | 50-59 years old (n=18) | 94.4% |  |  |  |
|  | 60-70 years old (n=20) | 90% |  |  |  |
| Employment | Employed (full or part-time) (n=35) | 80% | 4.225 | 3 | .238 |
|  | Student (full or part-time) (n=6) | 50% |  |  |  |
|  | Retired (n=14) | 92.9% |  |  |  |
|  | Disabled (not able to work) (n=6) | 100% |  |  |  |
|  | Unemployed (looking for work) (n=2) | 100% |  |  |  |
|  | Unemployed (not looking for work) (n=5) | 80% |  |  |  |
| T2D | Yes (n=36) | 86.1% | 1.556 | 3 | .669 |
|  | No (n=32) | 78.1% |  |  |  |
| Phone | iPhone (n=26) | 80.85 | 2.139 | 3 | .544 |
|  | Android (n=42) | 83.3% |  |  |  |
| Feedback 4 | | | | | |
| Age | 18-29 years old (n=15) | 80% | 29.735 | 27 | .326 |
|  | 30-39 years old (n=9) | 66.7% |  |  |  |
|  | 40-49 years old (n=6) | 83.3% |  |  |  |
|  | 50-59 years old (n=18) | 94.4% |  |  |  |
|  | 60-70 years old (n=20) | 95% |  |  |  |
| Employment | Employed (full or part-time) (n=35) | 82.9% | 4.123 | 3 | .248 |
|  | Student (full or part-time) (n=6) | 66.7% |  |  |  |
|  | Retired (n=14) | 100% |  |  |  |
|  | Disabled (not able to work) (n=6) | 100% |  |  |  |
|  | Unemployed (looking for work) (n=2) | 50% |  |  |  |
|  | Unemployed (not looking for work) (n=5) | 100% |  |  |  |
| T2D | Yes (n=36) | 94.4% | 7.162 | 3 | .067 |
|  | No (n=32) | 78.1% |  |  |  |
| Phone | iPhone (n=26) | 80.8% |  |  |  |
|  | Android (n=42) | 90.5% | 2.259 | 3 | .520 |
| Feedback 5 | | | | | |
| Age | 18-29 years old (n=15) | 73.3% | 56.645 | 36 | **.016** |
|  | 30-39 years old (n=9) | 77.8% |  |  |  |
|  | 40-49 years old (n=6) | 66.7% |  |  |  |
|  | 50-59 years old (n=18) | 88.9% |  |  |  |
|  | 60-70 years old (n=20) | 70% |  |  |  |
| Employment | Employed (full or part-time) (n=35) | 80% | 1.783 | 4 | .776 |
|  | Student (full or part-time) (n=6) | 50% |  |  |  |
|  | Retired (n=14) | 78.6% |  |  |  |
|  | Disabled (not able to work) (n=6) | 83.3% |  |  |  |
|  | Unemployed (looking for work) (n=2) | 50% |  |  |  |
|  | Unemployed (not looking for work) (n=5) | 100% |  |  |  |
| T2D | Yes (n=36) | 77.8% | 5.425 | 4 | .246 |
|  | No (n=32) | 75% |  |  |  |
| Phone | iPhone (n=26) | 76.9% | 1.769 | 4 | .778 |
|  | Android (n=42) | 76.2% |  |  |  |

Note: Significant p values are highlighted in bold. Feedback 1 = The length of the daily surveys on your smartphone; Feedback 2 = The length of baseline and follow-up questionnaire (The Qualtrics surveys sent by email); Feedback 3 = The frequency of the daily surveys on your smartphone; Feedback 4 = The type of phone sensor data being collected; Feedback 5 = The Beiwe app’s effect on my phone storage or battery Abbreviations: df = Degrees of Freedom; T2D = Type 2 Diabetes

Table S3

This table contains Pearson Chi-Square tests for relationships concerning the user experience questions “any other problem” and “any other feedback”.

| User experience: “any other problem” | | | | | |
| --- | --- | --- | --- | --- | --- |
| Answered any other problems vs didn’t answer | | | | | |
|  | Answered (23) | Did not answer (n=45) | χ | df | Sig (2-sided) |
| “Not a problem” to Feedback 1 | 91.3% | 88.9% | .522 | 2 | .770 |
| “Not a problem” to Feedback 2 | 78.3% | 82.2% | .945 | 3 | .815 |
| “not a problem” to Feedback 3 | 87% | 80% | 4.177 | 3 | .243 |
| “Not a problem” to Feedback 4 | 87% | 86.7% | 4.177 | 3 | .243 |
| “Not a problem” to Feedback 5 | 69.6% | 80% | 1.118 | 3 | .773 |
| Diabetes | 52.2% | 53.3% | 2.627 | 4 | .622 |
| Without diabetes | 47.8% | 46.7% |  |  |  |
| Answered positively/neutrally vs negatively | | | | | |
|  | Answered negatively (n=16) | Answered positively/neutrally (n=7) |  |  |  |
| “Not a problem” to Feedback 1 | 93.8% | 85.7% | .396 | 1 | .529 |
| “Not a problem” to Feedback 2 | 75% | 85.7% | .565 | 2 | .754 |
| “Not a problem” to Feedback 3 | 81.3% | 100% | 1.509 | 2 | .470 |
| “Not a problem” to Feedback 4 | 81.3% | 100% | 1.509 | 2 | .470 |
| “Not a problem” to Feedback 5 | 68.8% | 71.4% | 2.985 | 3 | .394 |
| Diabetes | 31.3% | 85.7% | 5.789 | 1 | **.016** |
| Without diabetes | 68.8% | 14.3% |  |  |  |
| User experience : “any other feedback” | | | | | |
| Answered any other feedback vs didn’t answer. | | | | | |
|  | Answered (n=14) | Did not answer (n=54) |  |  |  |
| “Not a problem” to Feedback 1 | 92.95 | 88.9% | 5.432 | 2 | .066 |
| “Not a problem” to Feedback 2 | 85.7% | 79.6% | .831 | 3 | .842 |
| “Not a problem” to Feedback 3 | 92.9% | 79.6% | 2.358 | 3 | .502 |
| “Not a problem” to Feedback 4 | 85.7% | 87% | 3.415 | 3 | .332 |
| “Not a problem” to Feedback 5 | 85.7% | 74.1% | 6.104 | 4 | .192 |
| Diabetes | 42.9% | 53.7% | .524 | 1 | .469 |
| Without diabetes | 57.1% | 46.3% |  |  |  |
| Answered positively/neutrally vs negatively | | | | | |
|  | Answered negatively (n=3) | Answered positively/neutrally (n=11) |  |  |  |
| “Not a problem” to Feedback 1 | 66.7% | 100% | 3.949 | 1 | **.047** |
| “Not a problem” to Feedback 2 | 66.7% | 90.9% | 1.131 | 1 | .287 |
| “Not a problem” to Feedback 3 | 66.7% | 100% | 3.949 | 1 | **.047** |
| “Not a problem” to Feedback 4 | 66.7% | 90.9% | 1.131 | 1 | .287 |
| “Not a problem” to Feedback 5 | 66.7% | 90.9% | 4.101 | 2 | .129 |
| Diabetes | 33.3% | 45.5% | .141 | 1 | .707 |
| Without diabetes | 66.7% | 54.5% |  |  |  |

Note: Significant p values are highlighted in bold. Feedback 1 = The length of the daily surveys on your smartphone; Feedback 2 = The length of baseline and follow-up questionnaire (The Qualtrics surveys sent by email); Feedback 3 = The frequency of the daily surveys on your smartphone; Feedback 4 = The type of phone sensor data being collected; Feedback 5 = The Beiwe app’s effect on my phone storage or battery. Abbreviations: df = Degrees of Freedom

Table S3

This table contains Pearson Chi-Square tests for relationships concerning hypothetical future use question.

| Hypothetical future use (n=65*) | | | | | | |
| --- | --- | --- | --- | --- | --- | --- |
| Hypothetical question (yes v no) | | | | | | |
|  |  | Yes (n=48) | No (n=17) | χ | df | Sig (2-sided) |
| Age | 18-29 years old | 18.8% | 29.4% | 10.182 | 9 | .336 |
|  | 30-39 years old | 10.4% | 23.5% |  |  |  |
|  | 40-49 years old | 10.4% | 5.9% |  |  |  |
|  | 50-59 years old | 29.2% | 17.6% |  |  |  |
|  | 60-70 years old | 31.3% | 23.5% |  |  |  |
| Gender | Male | 41.7% | 23.5% | 4.248 | 2 | .120 |
|  | Female | 58.3% | 75.6% |  |  |  |
| Education | Secondary school or less | 12.5% | 5.9% | 5.790 | 3 | .122 |
|  | Some post-secondary | 31.3% | 5.9% |  |  |  |
|  | Completed Bachelor’s | 27.1% | 41.2% |  |  |  |
|  | Masters or higher | 29.2% | 47.1% |  |  |  |
| Employment | Employed (full or part-time) | 43.8% | 70.6% | 7.647 | 1 | **.006** |
|  | Student (full or part-time) | 6.3% | 17.6% |  |  |  |
|  | Retired | 25.0% | 5.9% |  |  |  |
|  | Disabled (not able to work) | 16.7% | 0% |  |  |  |
|  | Unemployed (looking for work) | 8.3% | 5.9% |  |  |  |
|  | Unemployed (not looking for work) | 0% | 0% |  |  |  |
| Marital status | Single (never married) | 37.5% | 41.2% | .146 | 2 | .930 |
|  | Married or common-law partnership | 54.2% | 52.9% |  |  |  |
|  | Divorced, separated, or widowed | 8.3% | 5.9% |  |  |  |
| T2D | Yes | 64.6% | 11.8% |  |  |  |
|  | No | 35.4% | 88.2% | 14.012 | 1 | **<.001** |

Note: *3 participants who provided incongruent answers were removed from the 68 who answered the feedback questionnaire for the purposes of these analyses. Significant p values are highlighted in bold. Abbreviations: df = Degrees of Freedom

**Supplementary text file**

**Feedback questionnaire provided at study completion**

Thank you for taking part in our research. We would appreciate your feedback and opinions on the app and study.

Please indicate the extent to which each of these statements was a problem (or annoying/bothersome) for you during the study:

|  | Not a problem (1) | Minor problem (2) | Moderate problem (3) | Somewhat serious problem (4) | Serious problem (5) |
| --- | --- | --- | --- | --- | --- |
| The length of the daily surveys on your smartphone |  |  |  |  |  |
| The length of the baseline and follow-up questionnaires (the Qualtrics questionnaires sent by email) |  |  |  |  |  |
| The frequency of the daily surveys on your smartphone |  |  |  |  |  |
| The type of phone sensor data being collected |  |  |  |  |  |
| The Beiwe app’s effect on my phone storage or battery |  |  |  |  |  |

Other problems Please feel free to provide us with any other problems you experienced during the study (optional):

________________________________________________________________

________________________________________________________________

________________________________________________________________

________________________________________________________________

________________________________________________________________

other feedback Please feel free to provide us with any other feedback you may have (optional):

________________________________________________________________

________________________________________________________________

________________________________________________________________

________________________________________________________________

________________________________________________________________

We would like to ask you a hypothetical question (a question about a possible future).
 In the future, would you feel comfortable with your healthcare provider having access to information collected from your smartphone to better meet your needs and provide you with care?

- Yes (1)
- No (2)

If answered yes, the following question was displayed:

Could you tell us why? (optional)

________________________________________________________________

If answered no, the following question was displayed:

Could you tell us why not? (optional)

________________________________________________________________
