# Supplementary figures and images for: “Anything that would help is a positive development”: feasibility, tolerability, and user experience of smartphone-based digital phenotyping for people with and without type 2 diabetes
Source: BMC Digit Health. 2024 Sep 12;2(1):55. doi: 10.1186/s44247-024-00116-6 (PMC11390910; doi:10.1186/s44247-024-00116-6)

Figure S1: Data collection method tolerability

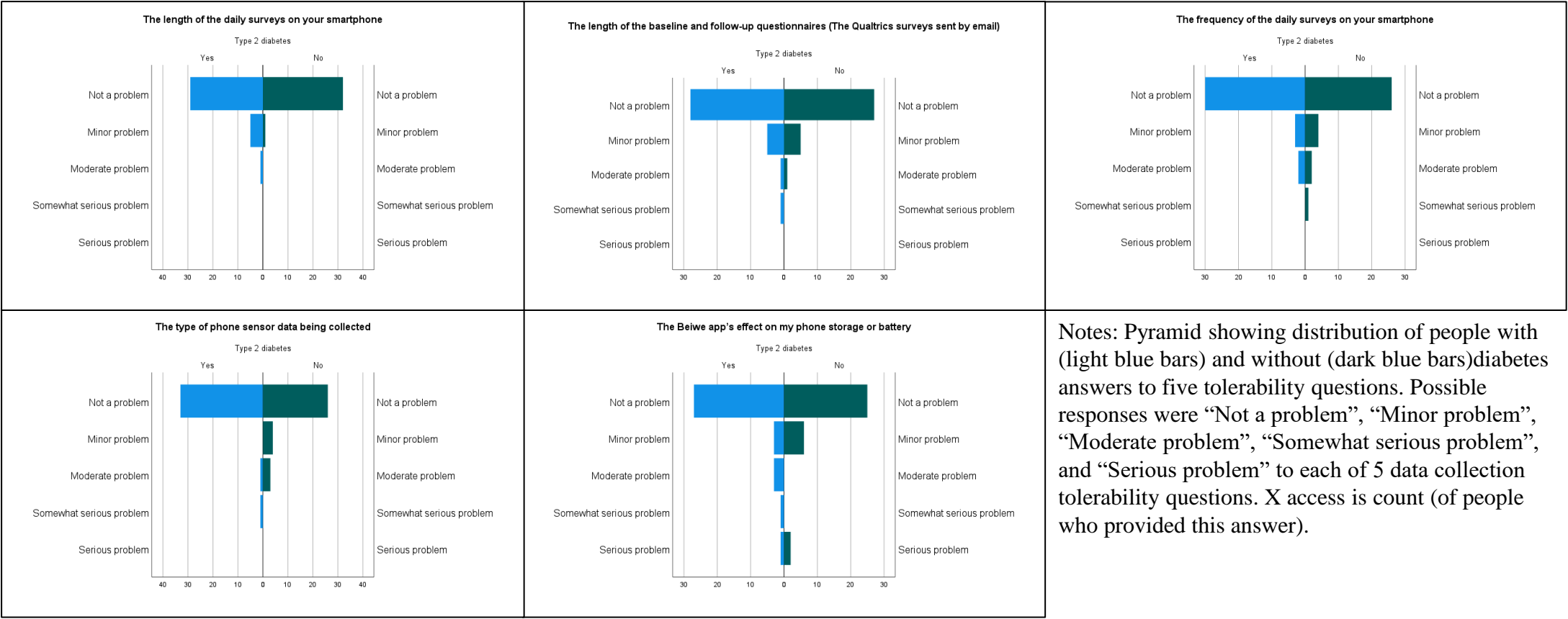

Supplement: Supplementary file 2 — Supplementary Material 2. [file 44247_2024_116_MOESM2_ESM.pdf]
